# HPV vaccination in women aged 27 to 45 years: what do general practitioners think?

**DOI:** 10.1186/1472-6874-14-91

**Published:** 2014-07-30

**Authors:** Danielle Mazza, Katja Petrovic, Cathy Grech, Naomi Harris

**Affiliations:** 1 Department of General Practice, School of Primary Health Care, Monash University, Building 1, 270 Ferntree Gully Road, Notting Hill, Victoria 3168, Australia

**Keywords:** Human papillomavirus, Vaccination, General practice, Attitudes, Beliefs, Barriers

## Abstract

**Background:**

Although the Human Papillomavirus (HPV) vaccine is registered in Australia for females aged 9 to 45 years, females aged 27 to 45 years have shown limited vaccine uptake. Our study explored general practitioners’ (GPs) views concerning HPV vaccination of females in this age group, with particular focus on the barriers and the facilitators to the delivery of the HPV vaccine.

**Methods:**

Semi-structured telephone interviews were conducted with 24 randomly selected general practitioners from metropolitan Melbourne. Questions were based on a theoretical framework that explained the barriers and facilitators to professional behaviour change.

**Results:**

According to the GPs, the major barriers to the uptake of the HPV vaccine included the cost of the vaccine, time constraints, and the three-dose schedule. Other barriers that were identified included GPs’ and patients’ beliefs that females in this age group were at low risk of contracting HPV, lack of awareness about the vaccine, and uncertainty about the benefits of this vaccine for females in this age group. In contrast, the facilitators that were identified included the availability of the vaccine on site, the availability of vaccine clinics or nurses for administering the vaccine, the availability of information related to the vaccine either on site or online, and positive opinions from experts in the field.

**Conclusions:**

Our study has identified some of the barriers and facilitators to the delivery and uptake of the HPV vaccine in females aged 27 to 45 years, as perceived by GPs. Further studies should be conducted to determine which of these should be targeted or prioritised for intervention. The views of women in this age group should also be considered as these would also be influential in designing effective intervention strategies for improving the delivery and uptake of the HPV vaccine.

## Background

Infection with the human papillomavirus (HPV) is the most significant risk factor for cervical cancer, the second most common cancer in women [1]. There are currently two vaccines against HPV that are available in Australia: Gardasil® (Merck & Co., Whitehouse Station, NJ USA) and Cervarix® (GlaxoSmithKline Biologicals, Rixensart, Belgium). Both vaccines demonstrate a high level of efficacy against HPV types 16 and 18 [2], which are classified as “high oncogenic risk” [3]. In Australia, Gardasil® is registered for use in females aged 9 to 45 years and in males aged 9 to 26 years [4], while Cervarix® is registered for use in females aged 10 to 45 years, but not in males [4].

In April 2007, a government-funded universal HPV vaccination program was introduced in Australia [5], the first country to do so. Initially under this program, females aged 12 to 26 years received the vaccine free of charge. School-based immunisation programs targeted girls aged 12 to13 years, and free vaccines were provided to general practice clinics to immunise females aged up to 27 years. Free school-based vaccination for girls aged 12 to 13 years still continues to this day; however, from December 2009, females aged 13 years and over incurred a private cost of approximately AUD$150 for each of the three vaccines required to complete the course (a total cost of AUD$450) [5]. In 2012, a similar HPV vaccination program for boys aged 9–18 years of age was also introduced in Australia [6,7].

Initial vaccination estimates suggest a high rate of HPV vaccine uptake in Australia, particularly through the school-based immunisation programs where a one-dose coverage of greater than 80% and a three-dose coverage of approximately 70% has been achieved [5,8]. However, the uptake in females aged up to 26 years who had access to the vaccine free of charge up until December 2009, but were not part of the school-based immunisation programs, may not be as high. One Australian study reported that only 58% of females aged 26 years and under had received at least one injection, with even fewer (26.9%) having completed the course [9].

Up to two-thirds of females aged 24 to 45 years are estimated to potentially be susceptible to all four HPV types present in Gardasil® (6, 11, 16, and 18) [10], yet very little is known about HPV vaccine uptake in this population [9,11]. A second peak of HPV prevalence has also been demonstrated in the fourth and fifth decades of life [12], which could be prevented by HPV vaccination. Although information on the efficacy of HPV vaccines in this age group is limited, one study has reported that Gardasil® is highly effective in preventing HPV-related diseases in females aged 15 to 45 years who were HPV naïve [13]. Such data is not yet available for Cervarix®; however, immunogenicity and safety data from a clinical trial indicate that this vaccine can induce a robust and persistent immune response in females aged 26–55 years [14].

In Australia, HPV vaccination of females aged 27 to 45 years primarily occurs in general practice, but little is known about the barriers to and facilitators of the delivery of the HPV vaccine in this population because previous studies have mainly focused on those aged 26 years and under [15-17]. Also, most studies that examined the beliefs of general practitioners (GPs) toward HPV vaccination were conducted outside of Australia [18-20], which limits the generalisability of the results to an Australian population. Consequently, the aim of our study was to investigate the barriers and facilitators to HPV vaccination in females aged 27 to 45 years in Australia, as perceived by GPs.

## Methods

This study was approved by the Monash University Human Research Ethics Committee and informed consent was obtained from all participants whose details were deidentified to maintain confidentiality.

To obtain a range of views that reflected diverse general practices, purposive sampling was used to recruit GPs. Letters of invitation were sent to a random sample of 400 GPs located in metropolitan Melbourne, Australia who were listed in the Australasian Medical Publishing Company database of Australian medical practitioners. An incentive of AUD$200 was offered for participation in the study. After two weeks, reminder letters were sent to GPs who had not yet responded. Of the 400 GPs selected, 44 showed interest in participating; however, only 24 GPs could be contacted within a reasonable timeframe for an interview.

Semi-structured telephone interviews were conducted by KP between November and December 2010, and the interview questions were based on the theoretical domains framework (TDF) [21] (Table 1). The TDF has been used in a number of qualitative studies to investigate the barriers and facilitators to the implementation of evidence-based practice in various health care settings [22-25]. Telephone interviews were conducted because of the difficulty in visiting some of the GPs due to their varying locations. The interviews were recorded using a digital voice recording device and then transcribed, with each transcript reviewed by the interviewer to ensure accuracy. The verified transcripts were imported into NVivo qualitative data analysis software (Version 7, QSR International Pty Ltd, Burlington, MA, USA) and a deductive process of thematic analysis was used to classify responses within themes, which were subsequently coded according to the domains of the TDF (Table 1) [21]. Transcripts were coded by two independent reviewers, and any disputes in coding were resolved through discussion and resolution.

**Table 1:** Interview questions and their corresponding construct domains

**Construct domain**	**Question**
Knowledge	What do you know about the HPV vaccine?
	Do you think there are any gaps in your knowledge of the vaccine?
	What do you know about HPV vaccination guidelines for women aged 27 to 45?
	How efficacious do you think the vaccine is in this age range?
	How strong do you think the evidence for vaccinating women in this age range is?
Skills	How easy or difficult do you find performing HPV vaccination in your context?
	How competent are you in administering the vaccine?
Social/professional role and identity	Do you feel a professional responsibility to encourage HPV vaccination? Why/why not?
	From an ethical standpoint, do you feel an ethical obligation to encourage HPV vaccination? Why/why not?
	Are there any other professional standards or guidelines that might conflict with recommending the HPV vaccine?
Beliefs about capabilities	How confident are you counselling women about the HPV vaccine?
	How confident are you in dealing with the issues inherent in HPV vaccination?
Beliefs about consequences	How beneficial do you think it would be for women in the 27 to 45 year age range to receive the vaccine?
	Do you believe the benefits of HPV vaccination outweigh the costs or not?
	Are there any side effects of HPV vaccination that you are concerned about?
Motivation and goals	Are there any particular incentives for you to recommend or administer the HPV vaccine? If so, what are they?
	How important do you believe HPV vaccination is?
	Is HPV vaccination a priority in your practice? Why/why not?
Memory, attention and decision processes	What sorts of things prompt or remind you to bring up the HPV vaccine with patients?
	Do you routinely recommend the HPV vaccine to women in the 27 to 45 year age range?
	Have there been times when you should have discussed HPV with a patient but didn’t?
Environmental context and resources	Are there other tasks or stressors which may dissuade you from taking the time to discuss HPV with patients? If so, what are they?
	Is there anything in your work environment that has in the past made it hard for you to administer the vaccine? If so, what?
	Does your practice contain all the necessary resources for providing information to women about the HPV vaccine?
	Does your practice contain all the necessary resources for administering the vaccine?
Social influences	Have you ever encountered any strong opinions on the HPV vaccine?
	Has there been any point at which you felt pressure from other people to encourage, or not encourage, HPV vaccination?
Emotion	Do you consider yourself to be emotionally motivated to administer the HPV vaccine to women in the 27 to 45 year age range?
	Are there any feelings, positive or negative, that might arise when you are counselling women about the HPV vaccine?
Behavioural regulation	What would need to be done in order to increase HPV administration at your practice?
	How willing would you be to make these changes?
	How long would these changes take?
	How could these changes be maintained over the long term?
Nature of the behaviour	How regularly do you initiate discussion about the HPV vaccine with women aged 27 to 45?
	What kinds of questions would you ask patients before deciding to recommend the HPV vaccine?

## Results

The GPs (n = 24) in this study comprised of 12 males and 12 females, with a mean age of 49 years (age range = 34–75 years). Data saturation was achieved after 15 interviews. The three principal domains described by GPs as being barriers and/or facilitators to HPV vaccination in females aged 27 to 45 years were: (1) environmental context and resources, (2) social influences, and (3) beliefs about consequences.

### Environmental context and resources

This domain identified barriers such as vaccine cost, time constraints, the GP’s physical work environment, the three-dose schedule, and the lack of availability of the vaccine on site.

### Cost

For the majority of GPs, a major barrier to HPV vaccination uptake was the cost of the vaccines to the patient, although most GPs said that this would not prevent them from discussing the vaccine.

“The most important problem is first of all, ‘how much is it?’ I tell them and their jaw drops”. (GP#15, female, age unknown)

Several GPs also indicated that patients were not willing to spend AUD$450 as they did not view HPV vaccination as a high priority.

“They seem to have enough money for everything else but their health care. So, now that is a real barrier to people having the vaccine”. (GP#7, male, 75 years old)

### Time constraints

The majority of GPs believed there was not enough time to address HPV vaccination during a consultation, and it was often sidelined because of other competing priorities.

“There's so many other things competing for our attention. You know, we’re asked to promote diabetes management and we’re asked to promote smoking cessation and things like that so there's so many other things that we’re trying to fit in”. (GP#17, male, 37 years old)

GPs indicated that they would be more inclined to discuss HPV vaccination if they were not so “time poor”. They were also more likely to raise it during consultations where Pap smears, sexual health or women’s health, contraception, sexually transmitted infections, or other vaccines were discussed.

“If you’re looking at heart problems, you talk about exercise. If you’re looking at sexual health, you talk about paps and HPV vaccinations”. (GP#4, male, 58 years old)

### GPs’ physical work environments

GPs’ physical work environments were either a barrier or an enabler to HPV vaccination depending on whether the vaccine was available on site, whether there were established routines such as vaccine clinics, and whether nursing assistance was available for vaccine administration.

“We’ve got the vaccines on site and we sell the vaccines and so there is ease of accessing the vaccine and delivering it”. (GP#16, female, 39 years old)

GPs also believed that the availability of sufficient information regarding HPV vaccination for patients and easier access to relevant information online would facilitate the delivery of the HPV vaccine to women aged 27 to 45 years.

### Inconvenience

Another major barrier to HPV vaccination was the three-dose schedule. GPs stated that patients were often reluctant to return for further consultations to complete the vaccination schedule. Similarly, requiring patients to go to the pharmacy to purchase the vaccine and then return at a separate time to get vaccinated was also viewed as a barrier by GPs who did not have the vaccine available on site. GPs felt that these inconveniences contributed to the low vaccine uptake.

### Social influences

This domain captured the role of patient perceptions and the influence of other peers and professionals on the delivery and uptake of the HPV vaccine.

### Patients’ perception of risk

According to GPs, a barrier to the uptake of the HPV vaccine in females aged 27 to 45 years was the patients’ perception of being at low risk of contracting sexually transmitted infections.

“They felt that they actually, they didn’t need to have it, because they were in a stable heterosexual relationship that they’d been in for 20 years, and they felt that their risk of getting anything was extremely low”. (GP#20, female, 49 years old)

Some GPs stated that they experienced negative reactions when raising the issue of sexually transmitted infections with patients in long-term relationships, with some patients becoming upset or insulted at suggestions that their relationship was not monogamous or stable.

### Patients’ awareness

GPs indicated that they were highly influenced by their patient’s agenda; any discussions regarding HPV vaccination depended on whether or not patients brought up the topic. However, GPs also felt that HPV vaccination was no longer on their patients’ agendas because it was no longer actively promoted in the wider community.

“When it first came out, as I said, we talked a lot more about it because people had read about it in the press and it was quite topical”. (GP#22, female, 44 years old)

### Opinion leaders

Positive opinions and information supporting HPV vaccination were perceived as a facilitator to the delivery of the HPV vaccine, especially if those opinions came from experts in the field.

“Usually when…makes a recommendation, I think, she is quite well respected in her field and I think you'd be foolish not to listen. Certainly I was encouraged to hear that someone of her calibre or standing in our profession felt it was a very good idea and we should be encouraging people to have it, so that definitely swayed my view”. (GP#22, female, 44 years old)

### Beliefs about consequences

This domain captured GPs’ beliefs on the benefits of HPV vaccination in females aged 27 to 45 years.

### GPs’ perception of patient’s risk of acquiring HPV

Another barrier to the delivery of the HPV vaccine to females aged 27 to 45 years was GPs’ belief that these women were at a lower risk of contracting HPV because they were more likely to be in stable, lifelong, monogamous relationships. GPs indicated that they were much more likely to discuss HPV vaccination with women who were not in a stable relationship, had just started a new relationship, gave an indication of multiple or changing partners, or mentioned that they had recently separated or divorced.

“I’d discuss it in categories of women who I thought were at risk, let’s put it that way. But people in the stable one to one relationship I doubt if I would bother discussing it to be quite honest”. (GP#7, male, 75 years old)

Several GPs believed that regular Pap smears for women in this age group were more important than receiving the vaccine, while other GPs did not feel the need to suggest vaccinating and would merely provide the patient with information and respect their decision.

### Uncertainty about consequences

A barrier to the delivery of the HPV vaccine by GPs was their uncertainty about the benefits of this vaccine in females aged 27 to 45 years. Many GPs voiced concerns that the vaccine had not been adequately tested in this age group and, therefore, the benefits of vaccinating them were unclear.

“The efficacy of the vaccine seems to wane, so even if you vaccinate an older woman, they’re not getting quite as good an immune response”. (GP#1, female, 46 years old)

Several GPs stated that there were no clear guidelines about recommending this vaccine to females in this age group and the circumstances under which it would be most appropriate.

“I’m not sure that there are very clear guidelines about that”. (GP#14, female, 41 years old)

“I don’t believe that there’s anyone who says that they know what you should do with that group”. (GP#19, male, 50 years old)

Consequently, many GPs were reluctant to recommend or administer the HPV vaccine to females in this age group.

Many GPs also believed that the risk amongst this population of contracting HPV varied and, therefore, vaccination should be determined individually and weighed against the financial cost to the patient. However, other GPs were confident that many females aged 27 to 45 years would derive significant benefit from HPV vaccination, with some believing that the evidence for vaccinating women of this age was very strong.

“I don't think there's any question about the efficacy of the vaccine in my mind, I think the vaccine is good, I think the trials have substantiated its efficacy so I'm happy to give it”. (GP#10, male, 59 years old)

Side effects were not a major concern for GPs with all stating that they believed the vaccine was safe and that they had not seen any major adverse reactions as a result of HPV vaccination.

## Discussion

Our study explored GPs’ perception of HPV vaccination in females aged 27 to 45 years. Using the TDF, we identified the barriers and facilitators to the delivery and uptake of this vaccine. The major barriers included the cost of the vaccine, time constraints, and the three-dose schedule. Other barriers that we also identified included GPs’ and patients’ beliefs that women in this age group were at low risk of contracting HPV, lack of awareness about the vaccine, and uncertainty about the benefits of this vaccine for this age group. In contrast, the facilitators that were identified included the availability of the vaccine on site, the availability of vaccine clinics or nurses for administering the vaccine, the availability of information related to the vaccine either on site or online, and positive opinions from experts in the field who support the use of the vaccine.

A major barrier to the delivery and uptake of HPV vaccination was the cost of the vaccine, which was also reported in a previous study that evaluated the early implementation of the national HPV vaccination program for young adult women in Australia [16]. This issue is not unique to Australia as a previous study from the US also reported the cost of the HPV vaccine as being a barrier to its uptake in young adult women [26]. Cost modelling studies have also shown that the cost benefit of HPV vaccination becomes less favourable with increasing age [13]. In the absence of a subsidy from the Australian government, it seems likely that recommending HPV vaccination to women in this age group will be determined by GPs on a case-by-case basis.

Another major barrier to the delivery of the HPV vaccine was the time constraints faced by GPs, which was also reported in previous HPV vaccination studies [16,27]. GPs noted that consultations of approximately 15 minutes were only adequate for addressing the patient’s immediate health issues, which deprives them of the opportunity to discuss HPV vaccination. Our results, however, may only be relevant in the Australian general practice context given that there is no registered patient population. In this context, preventive care is often done opportunistically at the end of a patient-driven consultation. Consequently, one of the key priority areas of Australia’s National Primary Health Care Strategy [28] is the development of systematic approaches for the delivery of preventive care activities.

GPs also stated that the three-dose schedule was a major barrier to the delivery and uptake of the HPV vaccine. Completing the HPV vaccine schedule was previously reported as an issue in another study because following up patients and asking them to return to the clinic to receive the second and/or third dose was often difficult [16]. A previous study also reported that women who had not completed the vaccine schedule were unlikely to do so because they simply forgot or had no time to return to the clinic [9]. One way to partially address the difficulty of following up patients is for general practices to incorporate a recall system into practice software or employ practice nurses to contact patients. Many of the GPs in our study believed that the availability of vaccine clinics or nurses at their practices could facilitate the delivery and uptake of the HPV vaccine.

Another barrier that we identified was the uncertainty amongst GPs about the benefits of vaccinating women in this age group. These uncertainties stemmed from GPs’ beliefs that women of this age group were at low risk of contracting HPV and the perceived lack of efficacy research and clear guidelines for this patient group. GPs indicated these women also believed that they were at low risk of HPV infection, which contributed to the low vaccine uptake. Patient perceptions of low risk have been observed in previous studies with younger populations [29,30], and may be even more pronounced in women who are in long-term, monogamous relationships [31]. GPs’ beliefs regarding the lack of efficacy data for this age group is not surprising given the paucity of publications regarding this at the time these interviews were conducted. However, since 2010, one study has reported that Gardasil® is highly effective in preventing HPV-related diseases in females aged 15 to 45 years who were HPV-naïve [13], but less effective in those who may have had a previous HPV infection [32]. These favourable results may convince GPs of the benefits of promoting HPV vaccination in older women even though Australian guidelines do not recommend HPV vaccination in all females aged 19 years and over because of the likelihood of exposure to at least one HPV type, which would reduce the efficacy of the vaccine [4]. Consequently, GPs will need to consider the benefits of HPV vaccination in older women by taking into account previous exposure and the risk of future exposure to HPV [4].

Potential facilitators to the uptake of the HPV vaccine that were identified by GPs included the availability of the vaccine on site, the availability of information related to the vaccine either on site or online, and positive opinions from experts in the field. The availability of the vaccine on site would certainly be more convenient for patients, but they would still be required to attend further consultations to complete the vaccine schedule. Improving the availability of and access to information about HPV vaccination is vital as many women believe that they do not have sufficient information at their disposal to allow them to make an informed decision about whether or not to get vaccinated [9]. This information, together with positive opinions from experts, may facilitate the uptake of the HPV vaccine because of the strong link between high HPV-related knowledge and greater intentions to vaccinate against HPV [33,34].

Our study is limited by the small number of GPs who were interviewed. Although a financial incentive was offered, the GPs in our study volunteered to participate and, therefore, may represent a subgroup of GPs who have a higher level of interest in HPV vaccination compared with GPs who were randomly selected. This potential bias may limit the generalisability of our findings; however, we found that some of the barriers reported in our study were also reported in similar studies from other countries [26,35-37], which supports the relevance of our study results to other countries even in the context of differing health systems.

Another limitation is the fact that all of the GPs in our study were from metropolitan Melbourne, Australia. Consequently, their responses may not be representative of the responses of GPs practicing elsewhere within Australia, particularly in rural areas. Conducting a similar study on a larger scale and also incorporating quantitative methodologies may provide a deeper understanding of the barriers that were identified in our study.

## Conclusions

Our study has identified some of the barriers and facilitators to the delivery and uptake of the HPV vaccine in females aged 27 to 45 years, as perceived by GPs. Further studies are necessary to determine which of these should be targeted or prioritised for intervention. We must also consider the views of women, particularly in relation to the cost of the vaccine versus the available data on efficacy for this age group and the risk of future exposure to HPV. Their views, along with those of GPs, are likely to influence the design and effectiveness of intervention strategies for improving the delivery and uptake of the HPV vaccine. These strategies should also consider the potential role of practice nurses in facilitating the delivery of the HPV vaccine especially to high-risk patients who should be actively targeted. Improving and increasing the availability of information on the HPV vaccine should also be considered in any intervention because of the importance of educating women (especially those who are deemed “high risk”) on its effectiveness in preventing certain types of cancer.

## Abbreviations

HPV: Human papillomavirus; GPs: General practitioners; TDF: Theoretical domains framework.

## Competing interests

The authors declare that they have no competing interests.

## Authors’ contributions

DM conceived the study and participated in the design of the study and the preparation of this manuscript KP participated in the design of the study, data collection, coding, and the preparation of this manuscript. NH and CG was involved in the coding of the participants’ responses and the preparation of this manuscript. All authors have read and approved the final manuscript.

## Authors’ information

DM (MD, MBBS, FRACGP, DRANZCOG, Grad Dip Women’s Health) is the Head of the Department of General Practice at Monash University. She is also the author of “Women’s Health in General Practice” (2nd edition published in 2011). Her current research interests include guideline development and implementation using patient-based strategies and closing evidence-practice gaps in preventive care, women's sexual and reproductive health, and cancer screening.

KP obtained her Masters degree in Psychology (Counselling) from Monash University in 2012. She is now working as a psychologist in private practice and school settings. She has also worked on other HPV-related articles and research studies concerning knowledge, attitudes and intentions relating to HPV vaccination.

CG received her Fellowship into the College of General Practitioners in June 2008. As well as her clinical role as a General Practitioner, CG has been working as a lecturer at Monash University since 2010. She is involved in medical student teaching and is a subject coordinator in the MBBS curriculum. Her areas of interest within General Practice include women’s health, sexual health and mental health, as well as medical education.

NH graduated with a MBBS from Monash University in 2003, and received her FRACGP, FARGP, and Grad Dip Rural GP in 2010. NH has been involved in the teaching program at Monash University since 2011. NH completed her Masters of Family Medicine, and is currently enrolled in her Masters of Sports Medicine. Clinically, she practises as a General Practitioner in various locations around Melbourne in the areas of women's health, paediatrics, mental health and sports medicine and is passionate about medical education.

## Pre-publication history

The pre-publication history for this paper can be accessed here:

http://www.biomedcentral.com/1472-6874/14/91/prepub
